# MiR-630 Inhibits Endothelial-Mesenchymal Transition by Targeting Slug in Traumatic Heterotopic Ossification

**DOI:** 10.1038/srep22729

**Published:** 2016-03-04

**Authors:** Yangbai Sun, Jiangyu Cai, Shiyang Yu, Shuai Chen, Fengfeng Li, Cunyi Fan

**Affiliations:** 1 Department of Orthopaedics, Shanghai Jiao Tong University Affiliated Sixth People’s Hospital, 600 Yishan Road, Shanghai, 200233, China

## Abstract

Heterotopic ossification (HO) is the abnormal formation of mature bone in extraskeletal soft tissues that occurs as a result of inflammation caused by traumatic injury or associated with genetic mutation. Despite extensive research to identify the source of osteogenic progenitors, the cellular origins of HO are controversial and the underlying mechanisms, which are important for the early detection of HO, remain unclear. Here, we used *in vitro* and *in vivo* models of BMP4 and TGF-β2-induced HO to identify the cellular origin and the mechanisms mediating the formation of ectopic bone in traumatic HO. Our results suggest an endothelial origin of ectopic bone in early phase of traumatic HO and indicate that the inhibition of endothelial-mesenchymal transition by miR-630 targeting Slug plays a role in the formation of ectopic bone in HO. A matched case-control study showed that miR-630 is specifically downregulated during the early stages of HO and can be used to distinguish HO from other processes leading to bone formation. Our findings suggest a potential mechanism of post-traumatic ectopic bone formation and identify miR-630 as a potential early indicator of HO.

Heterotopic ossification (HO) is a process by which cartilage or bone forms in soft tissues such as muscle, tendon and ligament in association with inflammation caused by severe injury, surgery or diseases^1^. Three types of HO have been described: fibrodysplasia ossificans progressiva (FOP), a pediatric metabolic disease characterized by the ossification of skeletal muscle; neurogenic HO, which occurs as a result of burns or neurologic injury; and traumatic HO, which occurs following injury to the tissues surrounding bones and joints. A variety of factors have been implicated in traumatic HO, including elbow dislocation, open injury, long wait time to surgery, and prolonged immobilization^2^,^3^. In addition to the acquired forms of HO, rare inherited disorders associated with extensive and severe HO have been described. There is currently no reliable method for the early detection of HO, which is critical to prevent the development of incapacitating conditions and permit effective intervention^4^,^5^.

In acquired, nonhereditary forms of HO, bone formation occurs as a secondary event and is associated with soft tissue trauma. In recent years, several studies have focused on identifying the cellular origin of the ectopic bone in HO. Hematopoietic cells or bone marrow progenitors were ruled out in bone marrow transplant studies in mice^6^,^7^. Traumatized muscle-derived mesenchymal stem cells (MSCs) were suggested as the putative osteoprogenitor cells that initiate ectopic bone formation in HO^8^ and vascular endothelial cells are likely sources of ectopic bone formation in patients with FOP, where approximately 50% of the cartilage and bone cells found in heterotopic lesions are of endothelial origin^2^,^9^. Endothelial-mesenchymal transition (EndMT), a newly identified process similar to epithelial-mesenchymal transition (EMT), is characterized by the loss of cell-cell adhesion and alteration in cell polarity leading to the development of elongated spindle-shaped cells^2^, and it is identified by the downregulation of endothelial markers such as CD31, VE-cadherin and von Willebrand factor (vWF) concomitant with the upregulation of mesenchymal markers such as N-cadherin and vimentin^10^,^11^. Newly formed mesenchymal cells in EndMT are highly invasive and motile, and have the capacity to give rise to various tissue types^10^. Medici *et al.* demonstrated that endothelial cells undergo EndMT to generate a MSC-like intermediate that can subsequently differentiate into osteoblast^9^.

MicroRNAs (miRNAs) are small, non-coding RNAs that regulate gene expression by specific binding to the 3′-UTR of target mRNAs, modulating expression by translational repression or mRNA destabilization^12^. Aberrant expression of many miRNAs has been found to be associated with EMT and EndMT in various diseases^13^,^14^,^15^. miRNAs have also been implicated in the regulation of osteogenic differentiation via different targets^16^,^17^.

In the present study, we used an *in vitro* model of EndMT based on the treatment of human dermal microvascular endothelial cells (HD-MVECs) with BMP4 and an *in vivo* model of HO and showed that the ectopic bone in traumatic HO is derived from the endothelium in the early phase. We elucidated a potential mechanism of post-traumatic HO involving miR-630 and the modulation of EndMT via the regulation of its target Slug. Comparative analysis of miR-630 expression in patients with HO and gender and age matched controls without HO after arthrolysis or with healing fractures was performed to reveal the role of miR-630 as an early indicator of HO, suggesting its potential clinical application for the early detection of HO.

## Results

### Endothelial origin of ectopic bone in traumatic HO

**Figure 1:**
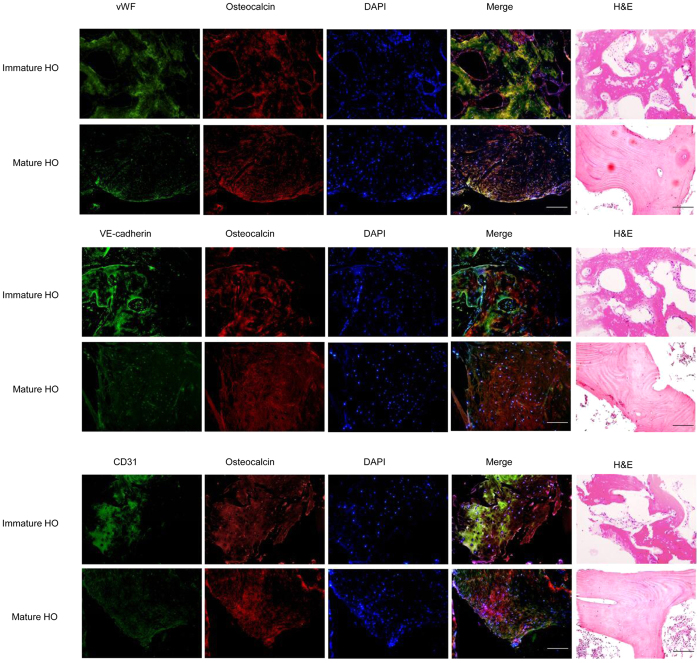
Ectopic bone in traumatic HO is of endothelial origin. Immunofluorescence analysis of lesional HO tissues from operation of trauma-induced HO patients was performed using antibodies specific for the endothelial markers vWF, VE-cadherin and CD31 and the osteoblast marker osteocalcin. H&E staining was perfromed for the new bone to identify the HO structure and cell composition. Immature HO: 1 month after ectopic bone is visible; Mature HO: 6 months after HO is visible. Representative images from one experiment out of three are shown. Scale bar, 50 μm. A more detailed immunofluorescenece analysis of negative controls can be found in Supplementary figure 1

We examined the origin of heterotopic bone by immunohistochemistry (IHC) analysis and hematoxylin-eosin (H&E) staining of ectopic bone samples obtained from patients who developed HO after trauma. We compared immature HO samples, representing 1 month after ectopic bone became visible, with mature HO samples collected 6 months after the appearance of HO. The H&E staining showed more compact in bone structure of mature HO. Moreover, double immunofluorescence staining for the endothelial markers vWF, E-cadherin or CD31 and the osteoblastic marker osteocalcin showed strong coexpression of endothelial and osteoblastic markers in both immature and mature HO. These results suggest that heterotopic bone formation arises from endothelial cells (Fig. 1).

### MiR-630 downregulated in immature HO

**Figure 2:**
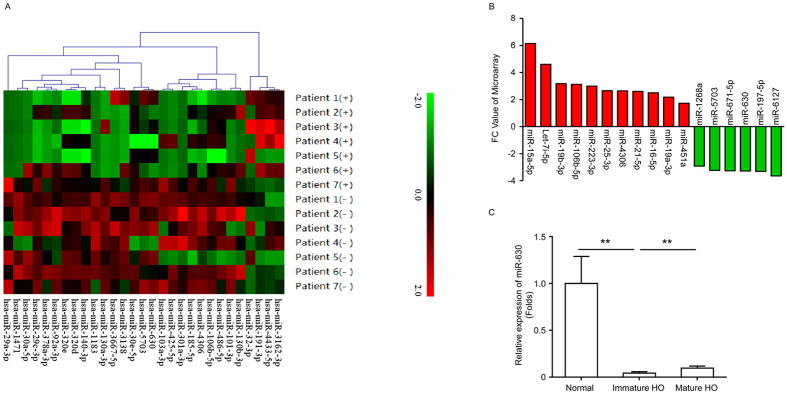
miR-630 is significantly downregulated in HO. (**A**) Microarray data from serum of patients with mature HO. (**B**) Significantly upregulated and downregulated miRNAs in patients after HO are shown (*P* of miR-630 < 0.01, unpaired *t* test). (**C**) Expression of miR-630 in the blood of normal people and patients with immature and mature HO (n = 65; Mean ± SEM; **P < 0.01; unpaired *t* test).

To explore the role of miRNAs in the regulation of HO formation via EndMT, we compared miRNAs expression profiles between patients with mature HO phase and immature HO phase. Microarray analysis of serum from seven patients with HO identified several up- and downregulated miRNAs in immature phase of HO compared to mature phase of HO. Of these, we focused on miR-630 based on previous studies showing the correlation of miR-630 with EMT and chondrogenic lineage differentiation^18^,^19^. Our results showed that miR-630 was downregulated in patients with HO (Fig. 2A,B). Further analysis of the expression of miR-630 showed approximately 20-fold and 10-fold downregulation of miR-630 in patients with immature and mature HO, respectively, suggesting that miR-630 may be an early marker of HO (Fig. 2C).

### Role of miR-630 and involvement of EndMT in HO

**Figure 3:**
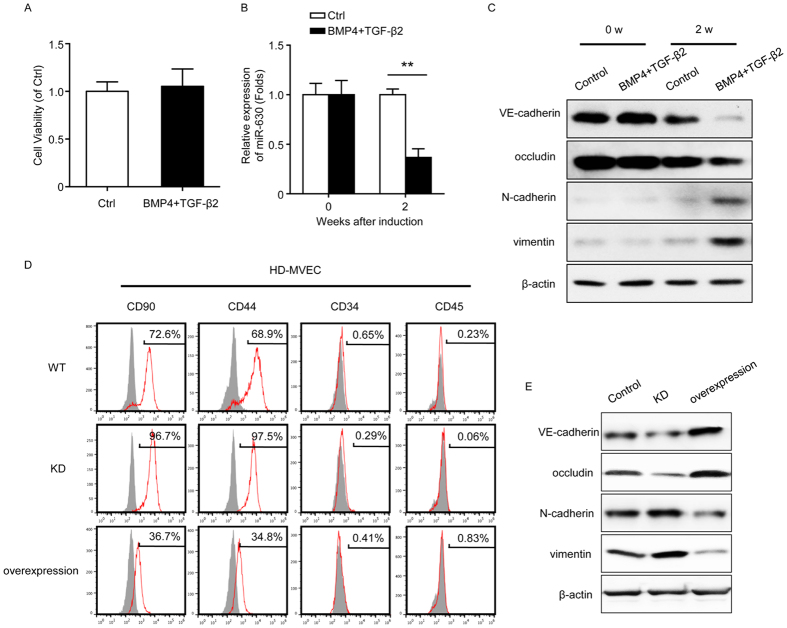
Effect of miR-630 on EndMT of HO. HD-MVECs were induced to undergo EndMT with or without BMP4 and TGF-β2 for 2 weeks. (**A**) MTT assay showed the cell viability of HD-MVECs after two-week culture. (**B**) The expression of miR-630 was measured by qRT-PCR (n = 3; Mean ± SEM; **P < 0.01; unpaired *t* test). (**C**) Expression of the endothelial markers VE-cadherin and occludin, mesenchymal markers N-cadherin and vimentin, were measured by western blot. (**D,E**) HD-MVECs were modified by miR-630 shRNA (KD) or miR-630 mimics (overexpression). Cells were induced to undergo EndMT with BMP4 and TGF-β2 for 2 weeks. The expression of MSC-like markers was measured by FCM on 10 days after induction (**D**) and the expression of EndMT markers was measured by western blot (**E**). (**B**) (n = 3; Mean ± SEM; **P < 0.01; unpaired *t* test). (**C–E**) Representative images from one experiment out of three are shown.

Bone morphogenetic protein 4 (BMP4) and transforming growth factor-β2 (TGF-β2) are known to induce activin-like kinase 2 (ALK2)-dependent EndMT, which is associated with heterotopic cartilage and bone formation^9^. We therefore induced EndMT in HD-MVECs by treatment with BMP4 and TGF-β2, and examined the expression of miR-630 and that of endothelial and mesenchymal markers to determine the involvement of the miRNAs and the cellular origin of ectopic bone in traumatic HO. MTT showed HD-MVECs exhibited no significant difference in the viability of HD-MVECs after two-week culture with or without BMP4 and TGF-β2 induction (Fig. 3A). miR-630 was significantly downregulated 2 weeks after EndMT induction in HD-MVECs, as determined by quantitative real-time PCR (qRT-PCR) (Fig. 3B). Western blot assessment of the expression of markers of EndMT showed that treatment with BMP4 and TGF-β2 for 2 weeks downregulated the endothelial markers VE-cadherin and occludin and upregulated the mesenchymal markers N-cadherin and vimentin, indicating the induction of EndMT in HD-MVECs (Fig. 3C). Flow cytometry (FCM) analysis of EndMT markers showed that Control HD-MVECs were positive for the MSC-like markers CD90 and CD44 and negative for the hematopoietic stem cell markers CD34 and CD45 2 weeks after the induction of mesenchymal markers, whereas those transfected with miR-630 mimics (overexpression) stained negative for CD90 and CD44, indicating that miR-630 inhibits EndMT (Fig. 3D). Western blot analysis confirmed these results, showing the upregulation of VE-cadherin and occludin concomitant with the downregulation of N-cadherin and vimentin in miR-630 overexpressing cells induced by BMP4 and TGF-β2, and the opposite pattern in KD cells (Fig. 3E). The formation of ectopic bone was examined in an *in vivo* model of HO induced by injection of Control or modified HD-MVECs into the hind limbs of mice.

### Effect of miR-630 on bone formation in HO

**Figure 4:**
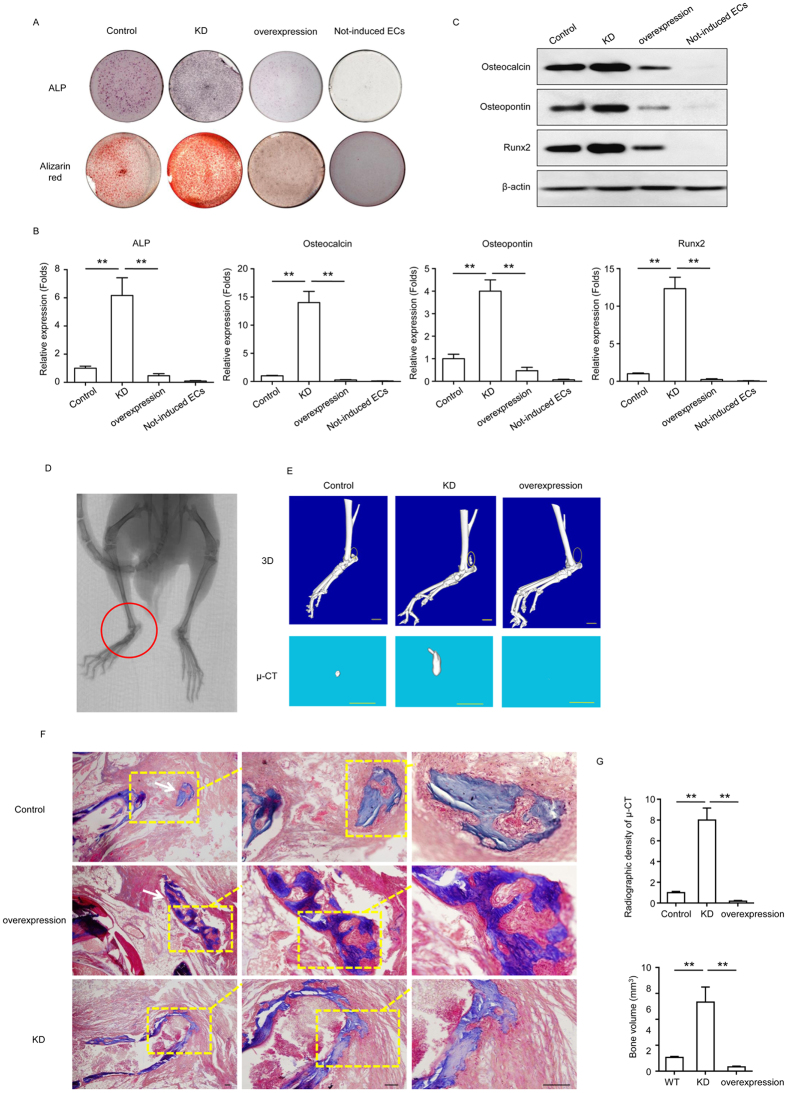
Effect of miR-630 on bone formation. HD-MVECs modified by miR-630 shRNA (KD) or miR-630 mimics (overexpression) were induced to undergo EndMT with or without BMP4 and TGF-β2 for 2 weeks and further induced for osteogenic differentiation for another 2 weeks. Cells and not-induced ECs were stained for ALP and analyzed for calcium deposition by Alizarin red S staining after induction. (**B**) Expression of ALP, osteocalcin, osteopontin and Runx2 by qRT-PCR. Data were normalized to the control group. (n = 3; Mean ± SEM; **P < 0.01; unpaired *t* test). (**C**) Western blot analysis of the expression of ALP, osteocalcin, osteopontin and Runx2. (**D**) HD-MVECs with or without modification were injected into the the region of the Achilles tendon of mice. X-ray image of HO (red circle) in a mouse implanted with Control HD-MVECs. (**E**) The hind himbs of mice implanted with HD-MVECs were examined by micro-CT, and representative images showing the 3D HO formation in each group 10 weeks after implantation are shown. Scale bar, 2 mm. (**F**) Ectopic bone formation as analyzed by Masson’s trichrome staining in the hind limbs of mice. Scale bar, 50 μm. (**G**) Quantification of radiographic density and bone volume. (**B,G**) (n = 3; Mean ± SEM; **P < 0.01; unpaired *t* test). (**A,G**) Representative images from one experiment out of three are shown.

To further examine the role of miR-630 in ectopic bone formation in HO, BMP4 and TGF-β2-induced HD-MVECs treated as indicated were subjected to alkaline phosphatase (ALP) and alizarin red S staining, and the relative expression of markers of osteoblastic differentiation was examined. The results showed that miR-630 KD markedly increased ALP and alizarin red S staining (Fig. 4A), and significantly upregulated the expression of the osteoblastic markers osteocalcin, osteopontin and Runx2 at both the mRNA (Fig. 4B) and protein (Fig. 4C) levels, whereas miR-630 overexpression had the opposite effects, suggesting that miR-630 inhibits ectopic bone formation in HO. Figure 4D shows an X-ray image of HO in mice injected with BMP4 and TGF-β2-induced Control HD-MVECs. Mineral deposition in the hind limbs of KD mice and the inhibition of HO in miR-630 overexpressing mice were analyzed by micro computed tomography (micro-CT) examination of the 3D HO formation in the hind limbs of mice (Fig. 4E) and Masson’strichrome staining (Fig. 4F). Quantitative analysis of radiographic density and bone volume of HO showed that knockdown of miR-630 significantly increased and overexpression of miR-630 significantly inhibited both bone density and bone volume in a mouse model of HO (Fig. 4G). Taken together, these results indicate that miR-630 downregulation may contribute to HO associated with EndMT and confirm the involvement of endothelial cells and role of the acquisition of a MSC-like phenotype in traumatic HO.

### Slug as a target of miR-630

**Figure 5:**
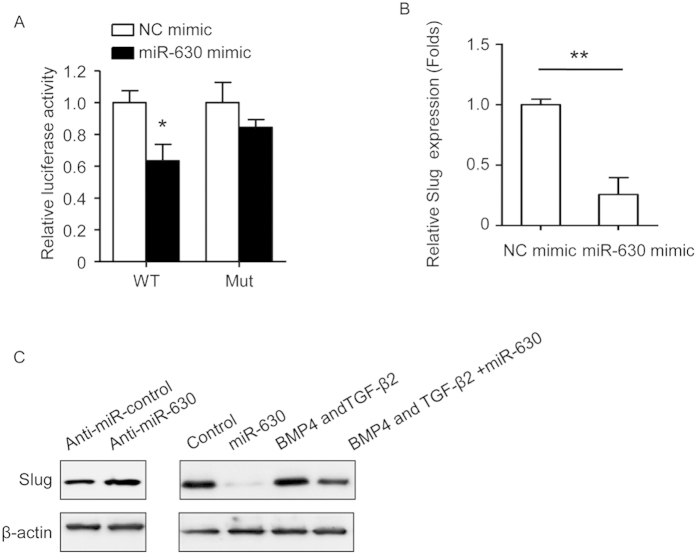
Slug is the direct target of miR-630. (**A**) LUC activity was determined 36 hours after transfection. Data were normalized to the LUC activity of negative control (NC) transfected cells. (**B**) qRT-PCR analysis of the expression of Slug in NC or miR-630 mimics transfected cells. (**C**) Western blot analysis of the protein expression of Slug after miR-630 overexpression or inhibition. Representative images from one experiment out of three are shown. (**B,C**) Results are Mean ± SEM from three independent experiments (n = 3; **P < 0.01; unpaired *t* test).

Because transcription factors of the Snail family of zinc proteins play a role in EMT and their interaction with specific miRNAs is thought to be important in the regulation of EMT^20^, we searched for potential targets of miR-630 associated with HO-related EndMT using the PicTar and TargetScan databases, and identified Slug as a miR-630 target. Cotransfection of HD-MVECs with LUC reporter constructs generated by cloning the Control or mutant 3′-UTR of Slug with miR-630 mimics showed that miR-630 overexpression significantly decreased the reporter activity of the Control but not that of the mutant Slug 3′-UTR (Fig. 5A). Ectopic expression of miR-630 significantly downregulated Slug mRNA expression compared to negative control mimic transfected cells (Fig. 5B). Western blot analysis showed that miR-630 overexpression markedly decreased Slug levels and suppressed the BMP4 and TGF-β2-induced upregulation of Slug (Fig. 5C).

### Rescue effect of Slug on the inhibition of EndMT by miR-630

**Figure 6:**
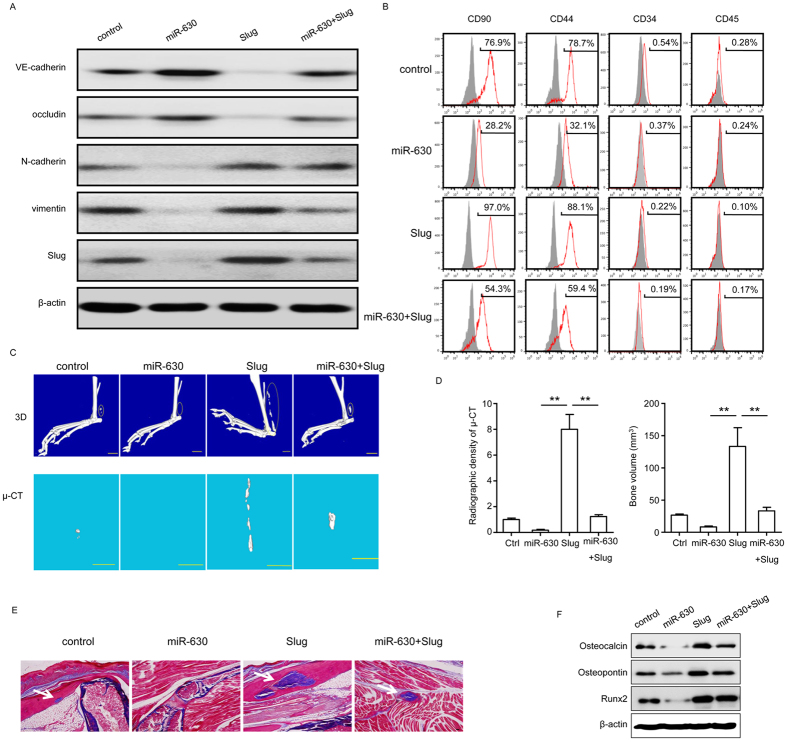
Slug overexpression rescued the inhibitory effect of miR-630 on EndMT. (**A,B**) HD-MVECs were induced to process EndMT with or without BMP4 and TGF-β2 for 2 weeks. Expression of EndMT markers VE-cadherin, occludin, N-cadherin and vimentin in each group was measured by western blotting (**A**). Expression of MSC-like markers was measured by FCM at 10 days after induction (**B**). (**C–F**) HD-MVECs were modified by miR-630 or Slug overexpression. After induction with BMP4 and TGF-β2 for 2 weeks, HD-MVECs with or without modification were injected into the hind limbs of mice. (**C**) Micro-CT examination of the limbs of mice implanted with HD-MVECs as indicated and representative images showing the 3D HO formation in each group 10 weeks after operation. (**D**) Quantification of radiographic density and bone volume (n = 3; **P < 0.01; unpaired *t* text). Results are mean ± SEM from three independent experiments. (**E**) Ectopic bone formation as determined by Masson’s trichrome staining in the hind limbs of mice. Scale bar, 50 μm. (**F**) Expression of ALP, osteocalcin, osteopontin and Runx2 by western blot. (**A**–**C,E,F**) Representative images from one experiment out of three are shown.

To further examine the effect of miR-630 regulation of Slug on EndMT associated with HO, the effect of ectopic expression of miR-630, Slug, or both miR-630 and Slug on the expression of EndMT markers and bone formation was examined in HD-MVECs. Overexpression of Slug reversed the miR-630-induced upregulation of endothelial markers and downregulation of mesenchymal markers (Fig. 6A), and restored the expression of mesenchymal markers by FCM (Fig. 6B). micro-CT examination of the 3D trabecular architecture showed that overexpression of Slug rescued the miR-630 inhibition of HO and mineral deposition in the hind limbs of mice as shown in representative 3D images and Masson’s trichrome staining (Fig. 6C,D).Quantitative analysis of radiographic density and bone volume confirmed these findings, showing that Slug significantly restored bone density and bone volume inhibited by miR-630 (Fig. 6E). In addition, Slug overexpression restored the levels of osteocalcin, osteopontin, and Runx2 downregulated by miR-630 (Fig. 6F). Taken together, these results indicate that the effect of miR-630 on the inhibition of EndMT and bone formation in HO is mediated by the downregulation of its target Slug.

### Serum miR-630 expression as a prognostic indicator of HO

**Figure 7:**
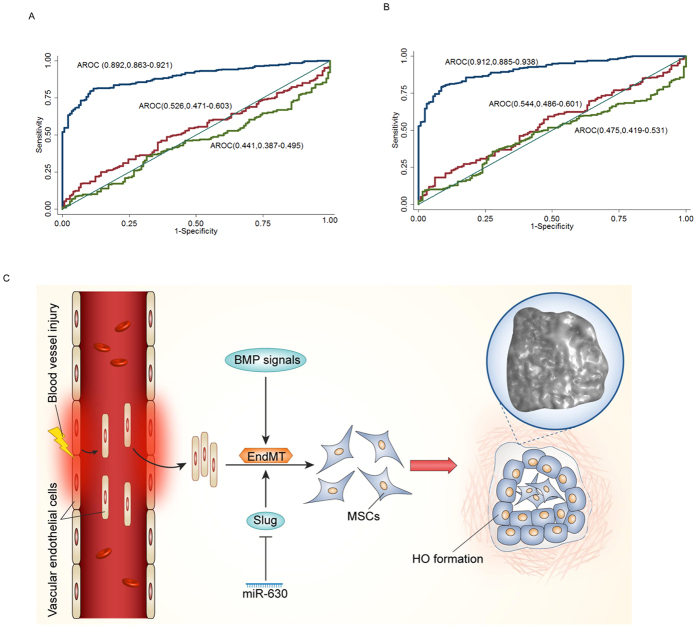
miR-630 expression as an early biomarker of HO. qRT-PCR analysis of miR-630 levels in the blood of post-traumatic HO patients, compared with patients who underwent arthrolysis operation without HO (Ctrl1) (**A**) and patients showing bone healing after bone fracture (Ctrl2) (**B**). The area under the ROC curves for detecting patients with HO from those who underwent arthrolysis operation without (ctrl1) (**A**) and patients showing bone healing after bone fracture (Ctrl2) (**B**) Blue line: Serum miR-630 at baseline; Red line: Serum miR-630 at 3-month follow-up; Green line: Serum miR-630 at 1-year follow-up. (**C**) miR-630 inhibits EndMT by targeting Slug in traumatic HO. After the blood vessels suffer injured, vascular endothelial cells are immediately released into muscle tissue and undergo EndMT in response to the BMP signals.miR-630 inhibits EndMT through mediating the expression of target gene Slug. These endothelium-derived MSC-like cells finally lead to new HO formation.

The effect of miR-630 on HO was further investigated by comparing the serum levels of miR-630 between patients with trauma-induced HO and age and gender matched controls without HO (Ctrl1) or patients showing bone healing after fracture (Ctrl2). At baseline, i.e. within 8 hours after injury, miR-630 expression was significantly lower in patients with HO compared to Ctrl1 patients who underwent arthrolysis without HO (0.93 ± 0.03 vs 2.58 ± 0.12, *P* < 0.05) and compared to Ctrl2 patients (0.93 ± 0.03 vs 2.51 ± 0.09, *P* < 0.05, Table 1). At the time when ectopic bone was visible, i.e. 3 months after surgery, miR-630 was also decreased in HO than in Ctrl1 (2.09 ± 0.09 vs 2.50 ± 0.14) and Ctrl2 (2.09 ± 0.09 vs 2.49 ± 0.11, Table 1) but the difference did not reach statistical significance. The serum miR-630 level at 1-year follow-up after surgery was similar among three groups. During the first 8 h after injury, the AUC of miR-630 for identifying patients with HO from those without HO (Ctrl1) was 0.892 (95% CI, 0.863–0.921, Fig. 7A), and the AUC of miR-630 for detecting patients with HO from those with bone healing after injury (Ctrl2) was 0.912 (95% CI, 0.885–0.938, Fig. 7B). For identifying Ctrl1 patients, the serum miR-630 of 1.25 or lower corresponded to the point on the AUC curve at which sensitivity (88.4%) plus specificity (81.2%) were maximized, with a positive predictive value of 70.1%. Using miR-630 of 1.25 as the cut-off point, the sensitivity, specificity and positive predictive value for detecting Ctrl2 patients were 88.4%, 80.8% and 69.7%, respectively. At the time of 3-months and 1-year follow-up after the surgery, the AUC of miR-630 for detecting patients with HO from Ctrl1 and Ctrl2 patients did not show statistical significance (Fig. 7A,B).

## Discussion

Despite extensive research aimed at identifying the cellular origin and processes leading to HO occurrence, the mechanisms underlying the formation of ectopic bone remain unclear, and the cell population undergoing osteogenic differentiation in post-traumatic HO remains controversial. In addition, an effective biomarker for the early detection of HO in patients undergoing traumatic injury has not been identified to date. In the present study, we explored the source of osteogenic progenitors in post-traumatic HO during the early phase and elucidated a potential underlying mechanism involving the induction of EndMT and the miRNA mediated modulation of gene expression. A matched case control study showed the ability of miR-630 to specifically detect HO during the early stages, suggesting the potential use of miR-630 as an early clinical indicator of HO.

In the present study, IHC analysis of ectopic bone samples from patients with post-traumatic HO showed colocalization of endothelial markers with osteocalcin, a marker of osteogenic differentiation. Since osteoblasts from normal bone do not express these endothelial markers, these results suggest that osteoblasts in heterotopic lesions are of endothelial origin^9^. In animal models of FOP, endothelial precursors were shown to differentiate through an endochondral pathway and form heterotopic bone in response to constitutive BMP signaling, supporting a vascular endothelial origin of progenitor cells in HO^21^. Similarly, in a model of BMP4 and TGF-β2-induced HO, ectopic skeletal cells were shown to express VE-cadherin, suggesting the conversion of endothelial cells to MSC-like cells via EndMT, and their further differentiation into an osteoblastic phenotype^9^. However, a study that used lineage tracing and heterotopic ossification bioassays identified a progenitor cell population with osteogenic potential residing in the interstitium of skeletal muscle and showed that it was distinct from the endothelium, thus arguing against the role of endothelial cells in BMP2-induced HO^22^. Moreover, another study reported that the induction of HO was associated with muscle-resident mesenchymal or stromal cells which increased the expression of ALK1, the BMP-9 receptor^23^. Circulating hematopoietic derived cells with osteogenic potential were also suggested to function as osteogenic precursors in HO^7^. A recent study based on the detection of specific cellular markers in cell suspensions of traumatized muscle found that the most abundant subpopulations were CD29^+^ and CD34^+^, which had the greatest migratory capacity and expressed VE-cadherin, Tie2, and CD31 when cultured under differentiation conditions, suggesting an endothelial origin of ectopic bone in post-traumatic HO^24^. Although several cell populations have the potential to differentiate into osteogenic precursors in HO and the origin of progenitor cells involved in HO is still very debatable^22^,^23^, our present results support an endothelial origin of osteogenic precursors and the role of EndMT in the generation of a MSC-like intermediate with osteogenic potential during the early phase of HO formation.

The role of EndMT in post-traumatic HO was examined using a model of BMP4 and TGF-β2-induced EndMT. In addition, according to microarray analysis which identified miR-630 as a downregulated miRNA in patients with HO, and all filtered-out miRs (including miR-197, miR-671 ) which were tested by relative expression in the blood of normal people and patients with immature and mature HO, and the miR-630 was selected as a target for study. As it was showed that miR-630 was downregulated during early stage of HO, we further examined the role of miR-630 in BMP4 and TGF-β2-induced HD-MVECs and in a mouse model of HO *in vivo*. Our results showed that BMP4 and TGF-β2 induction downregulated miR-630 concomitant with the upregulation of mesenchymal markers and downregulation of endothelial markers, suggesting that miR-630 is involved in EndMT induction. The effects of miR-630 KD and overexpression on mesenchymal and endothelial marker expression, *in vivo* ectopic bone formation, and bone density and bone volume indicated that miR-630 inhibited EndMT and the associated ectopic bone formation. The association of miRNAs with EndMT has been demonstrated in specific conditions such as cardiac fibrosis and kidney fibrosis^13^,^25^,^26^,^27^. In addition, miRNAs implicated in osteogenic differentiation have been identified in previous studies^28^,^29^. Evidence of the involvement of miRNAs in HO is limited to the correlation between miRNAs and ectopic bone formation or alterations in miRNA expression in serum. A study that identified miRNA expression signatures during mesenchymal to chondrogenic differentiation showed significant increases in miR-92b, miR-140, miR-574-3p and miR-1231^29^. It was found that patients with traumatic injury had higher serum levels of miR-16 and miR-92a, which indicated that serum biomarkers might play a role in ectopic bone formation^30^.

The effect of miR-630 on the inhibition of EndMT and the induction of HO was mediated by the downregulation of the transcription factor Slug, which was identified as a target of miR-630. Ectopic expression of Slug reversed the miR-630-induced upregulation of endothelial markers and downregulation of mesenchymal markers in HD-MVECs and reversed the miR-630 inhibition of ectopic bone formation in animal models, as well as the related downregulation of osteoblastic markers. TGF-β2 and BMP4 stimulation induce the expression of Slug, which is among several transcription factors that promote EMT and the conversion of endothelial cells into MSC-like cells with the potential to differentiate into distinct lineages^9^. The involvement of miRNAs in EMT via the modulation of Slug expression has been studied mostly in cancer. Specific regulatory networks involving miRNAs and EMT related transcription factors have been described, such as the miR-203/Snai1feedback loop and its role in the regulation of EMT^31^, the inhibition of EMT by miR-1 and miR-200 via Slug^32^, and a p53/miR-34 axis that modulates EMT via the downregulation of Snail1 among others^33^. Although miR let-7 were not the found to have a statistical significance in miR analysis of our work, the anti-EndMT effects should also be emphasized in the EndMT process^34^. Our findings suggest a potential mechanism underlying the regulatory role of miR-630 in HO via the modulation of Slug expression and EndMT, and may thus lead to the potential identification of therapeutic targets and biomarkers in HO.

The early detection of HO is critical to enable adequate treatment to prevent the recruitment of progenitor cells during the early stages of HO formation. Currently, ALP is the most commonly used clinical indicator of HO^35^. However, its specificity is limited; therefore, the identification of a sensitive and specific indicator of HO is essential. To examine the potential role of miR-630 as a biomarker, we performed qRT-PCR analysis of serum samples of patients with post-traumatic HO, which showed that miR-630 was significantly downregulated early after the occurrence of trauma, and its expression remained low at the time of detection of ectopic bone formation, increasing progressively thereafter. These findings indicate that the levels of miR-630 could be an indicator of ectopic bone formation after trauma. Additional studies are necessary to confirm these findings in a larger population and at additional time points during the development of post-traumatic HO.

In conclusion, our results provide evidence of the endothelial origin of ectopic bone in the early phase of HO formation via the induction of EndMT and the potential differentiation of cells via a mesenchymal cell intermediate HO formation. We showed that miR-630 was downregulated in patients with HO and inhibited EndMT via the downregulation of its target Slug. Analysis of serum miR-630 levels revealed a distinct pattern of expression in response to trauma-related HO, providing potential biomarkers and therapeutic targets for the management of patients with post-traumatic HO.

## Methods

### Cell culture, EndMT induction and Osteogenic differentiation

HD-MVECs were purchased from Lifeline Cell Technology (Frederick, MD, USA). For EndMT induction, cells were cultured in endothelial growth medium-2 (Lifeline) with 10 ng/ml BMP4 and 5 ng/ml TGF-β2 (R&D Systems, Minneapolis, USA) for 2 weeks as described previously with some modifications^9^. For osteogenic differentiation, cells were further cultured in DMEM supplemented with 10% FBS, 10 nM dexamethasone, 100 mM L-ascorbic acid, and 10 mM β-glycerophosphate (Sigma-Aldrich, St Louis, Mo, USA) for another 2 weeks. Cell viability was analyzed using the MTT assay provided in a cell proliferation kit (MTT, Roche) was performed in 96-well plates according to the manufacturer’s instructions.

### FCM analysis

Cells were collected and stained with antibodies against human antigens such as CD90, CD44, CD34 and CD45 (BD Pharmingen, San Diego, CA, USA). Stained cells were washed and analyzed with a FACSAria cytometer (BD Biosciences, San Diego, CA, USA).

### Cell transfection and Plasmid construction

For the HD-MVECs transfection assay, the synthetic miR-630 mimic (Applied Biosystems, Shanghai, China), miR-630 inhibitor (Applied Biosystems), and their corresponding negative control (miR-NC and anti-NC) were transfected using by Lipofectamine2000 (Invitrogen Inc., Carlsbad, CA, USA), which were designed and synthesized by Invitrogen. The human miR-630 and Slug coding sequence were cloned into the lentivirusvectorp CDH (System Biosciences, Mountain View, Wi, USA) to generate stably transfected cell lines. 293T cells (The American Type Culture Collection, Manassas, VA, USA) were cotransfected with lentiviralvectorp CDH and packaging plasmid to generate lentivirus. The 293T cells supernatant was collected at 48 hours post-transfection. After viral titer determination, the lentivirus carrying miR-630 or Slug was used to infect cells.

### Immunofluorescence staining and H&E staining

Ectopic bone was fixed in 4% paraformaldehyde, permeabilized with 0.1% Triton X-100, blocked in 1% BSA, and incubated overnight with primary Abs (Abcam, Cambridge, UK). Subsequently, cells were incubated with fluorochrome-conjugated secondary Ab (Invitrogen), counter stained with DAPI (Roche Applied Science, Indianapolis, IN, USA), and observed under a fluorescence microscope (Nikon Eclipse E-400; Tokyo, Japan). The ecopic bone and sponge bone as negative control were cut into 6 μm sections. They were then deparaffinated, hydrated and stained with H&E.

### Dual-LUC reporter assays

The 3′-UTR regions of human Slug that contain the miR-630-binding sites were amplified using the following specific primers: Slug sense, 5′-CCG CTC GAG CAA AGA TAA AAT GAA AAG C-3′; Slug antisense, 5′-CCG ATA AGA ATT GCA CTT ATT CCC ATC TTT A-3′; Overlapping PCR was performed to mutate six bases of seed sequence in miR-630-binding sites, using additional two pair of primers: slug mut sense, 5′- CCG GAA TTC TTT TTA AAA GGA GGA AAA A-3′; Slug mut antisense, 5′- CCG GAA TTC TTA AAG CAC TAC AGG TAA TC-3′; miR-630 or mutated 3′-UTR was inserted into the psiCHECK-2 vector after amplified. Thirty-six hours after transfection, LUC activity was detected using the Dual-LUC Reporter Assay System (psiCHECK-2 vector; Promega, Madison, WI, USA) and normalized to Renilla activity.

### ALP and Alizarin red S staining

After 14 days of the induction of osteogenic differentiation, cells were washed with PBS and fixed with 4% paraformaldehyde for 30 min. For ALP staining, cells were stained with 5-bromo-4-chloro-3-indolyl-phosphate/nitro-blue tetrazolium solution (Sigma-Aldrich) for 45 minutes at 37 °C to visualize ALP activity. For Alizarin red, cells were stained with 2% Alizarin red S staining solution (pH 4.2; Sigma-Aldrich) for 30 minutes at 37 °C to visualize matrix calcium deposition.

### Mice

All animal experimental protocol (n = 42) were approved by the Animal Research Committee of Shanghai Jiao Tong University Affiliated Sixth People’s Hospital. Forty-two BALB/c-nu mice (n = 6 per group) were purchased from the Shanghai Laboratory Animal Center of the Chinese Academy of Sciences (Shanghai, China) and underwent lateral midpoint Achilles tenotomy through a posterior approach underaseptic condition. Modifed HD-MVECs and 200 μl growth factor-reduced Matrigel(BD Biosciences) were then injected into the the region of the Achilles tendon in hind limbs of mice.

### Micro-CT analysis

All mice groups were analyzed by micro-CT using Skyscan 1176 scanner (Sky scan, Kontich, Belgium) at 9 μm resolution 10 weeks after operation. New HO formation was evaluated though 3D images constructed by the InstaRecon/NRecon software (Skyscan). Besides new HO formation volume and bone mineral densities were also calculated. Each scan took 20 min, with the mice under isofluorane anesthesia. No complications were observed during the scans.

### Bone histomorphometric analysis

After taken the micro-CT analysis, mice were sacrificed and hind limbs of each groups were fixed in 10% neutral buffered formalin for 48 h, dehydrated in alcohol solutions, and embedded in methylmethacrylate (Technovit9100, Heraeus, Wehrheim, Germany). Longitudinal sections in a sagittal plane were cut at 10 μm by a SM 2500 Microtome (Leica, Bensheim, Germany). Masson’s Trichrome staining (BaSO Diagnostic Inc., Guangdong, China) was implemented to examine themorphology. Finally, the sections were mounted to observe the structural changes in the annulus fibrosus under a light microscope^36^.

### qRT-PCR

Total RNA was extracted from tissues or cells at indicated time points and was subsequently reverse-transcribed using AMV Reverse Transcription System (Takara, Shiga, Japan). qRT-PCR was performed using SYBR Green PCR mix on an ABI Prism 7900HT (Applied Biosystems, Foster City, CA, USA). (β-actin forward primer: 5′-TGT CCA CCT TCC AGC AGA TGT-3′, β-actin reverse primer: 5′-AGC TCA GTA ACA GTC CGC CTA GA-3′; ALP forward primer: 5′-TTG TGC GAG AGA AGG AGA-3′, ALP reverse primer: 5′-GTT TCA GGG CAT TTT TCA AGG T-3′; Osteocalcin forward primer: 5′-CTG ACA AAG CCT TCA TGT CCA A-3′, Osteocalcin reverse primer: 5′-GCG CCG GAG TCT GTT CAC TA-3′; Osteopontin forward primer: 5′-GTA TTG CTT TTG CCT TTG CCT GTT TGG-3′, Osteopontin reverse primer: 5′-TGA GCT GCC AGA ATC AGT CAC T-3′; Runx2 forward primer:5′-CGC CCC TCC CTG AAC TCT-3′, Runx2 reverse primer: 5′-TGC CTG CCT GGG ATC TGT A-3′; Slug forward primer: 5′-GGG GAG AAG CCT TTT TCT TG-3′, Slug reverse primer: 5′-TCC TCA TGT TTG TGC AGG AG-3′.

For miRNA quantitation, cDNA was reverse transcribed from the total RNA samples using specific miRNA primers from the TaqMan MicroRNA Assays and reagents from the TaqMan MicroRNA Reverse Transcription kit (Applied Biosystems). The cDNA was amplified by PCR using TaqMan MicroRNA Assay primers with the TaqMan Universal PCR Master Mix. The relative levels of miRNA expression were calculated from the relevant signals by normalization with the signal of U6 snRNA expression.

### Western blot analysis

Cells were directly lysed with RIPA containing protease and phosphatase inhibitors (Roche Applied Science) and proteins were separated by 10% SDS-PAGE after denaturation. Immunoblot analysis was performed by initial transfer of proteins onto polyvinylidenefluoride membranes using Mini Trans-Blot (Bio-Rad Laboratories, Richmond, VA, USA) and followed by a blocking step with 5% nonfat dried milk plus 0.1% Tween 20 for 2 hours at room temperature and exposed to primary antibodies diluted 1,000-fold that recognized Slug, osteocalcin, osteopontin, Runx2, VE-cadherin, occludin, N-cadherin, vimentin or β-actin overnight at 4 °C and subsequently washed. The blots were then incubated with a secondary Ab conjugated with Horse Radish Peroxidase diluted 5,000-fold for 1 hour at room temperature. Signals were detected by FluorChem E system (Alpha Innotech Corp, Santa Clara, CA, USA).

### Microarray and data processing

Total RNA was extracted using the TRIzol method (Invitrogen) according to the manufacturer’s protocol. MiRNA microarray analysis was performed using Exiqon’s microarray platform (version 8.0; containing 330 human miRNAs; Exiqon, Vedbaek, Denmark) in quadruplicate with LNA-modified oligonucleotide (mercury; Exiqon) capture probes. Slides were scanned using a Genepix 4000B laser scanner (Axon Instruments, Foster City, CA, USA). Artifact-associated spots were eliminated by software (TIGR spotfinder 3.1.1). Image intensities were measured as a function of the median of foreground minus background. Negative values and values below 50 were normalized to one. Additional data analysis was performed using Microsoft Excel with Significant Analysis of Microarrays (SAM) excel software with multiclass response dataset analysis. The data were normalized using the Limma package for statistical programming language R, version 2.5.1. Medians of four background corrected replicas for each miRNA capture probe were uploaded into the microarray analysis software for more advanced analysis.

### Study population and sample acquisition

We recruited 730 traumatic patients in the department of Orthopaedics at Shanghai Jiao Tong University Affiliated Sixth People’s Hospital. Cases were 146 patients with HO (53 women; age range, 9–60 years), and control 1 group (Ctrl 1) were 292 age- and sex-matched patients without HO after taking arthrolysis for the stiff elbow (106 women; age range, 9–60 years), and control 2 group (Ctrl 2) were 292 age- and sex-matched patients with fracture healing after open reduction internal fixation (106 women; age range, 8–60 years). HO was diagnosed independently by a radiologist and an orthopedic surgeon, who were blinded to the treatment which patients received. Exclusion criteria included affected bone metabolic diseases, renal dysfunction (serum creatinine >2 mg/dl), mental disorder, diabetes mellitus, hyperprolactinemia, rheumatoid arthritis, malabsorption syndromes, malignant tumors, hematological diseases, or a history of pathological fractures, or patients with medications of glucocorticoids, estrogens, thyroid hormone, parathyroid hormone, floride, bisphosphonate, calcitonin, thiazide diuretics, barbiturates, and antiseizure medication. Trained stuff (*n* = 730) administered a detailed questionnaire face to face. Samples of ectopic bone from patients with HO and serum from all participants of each group were obtained between October 2010 and November 2014. Venous blood samples were drawn for measuring serum level of miR-630 within 8 hours after injury, and 3 months and 1 year after surgery, respectively. Serum miR-630 level was measured using qRT-PCR. This study was approved by our ethics committees and was conducted in accordance with the principles of the Declaration of Helsinki.

### qRT-PCR analysis for miR-630 of serum

For qRT-PCR analysis of miR-630, TaqMan miRNA reverse transcription kit and a TaqMan miRNA assay kit (Applied Biosystems, Foster City, CA, USA) were used. Quantitative real-time PCR was performed on the ABI PRISM Sequence Detection System (AppliedBiosystems). The mean C_t_ values of each sample were determined from triplicate reactions. The relative expression level of miR-630 examined was calculated by log_2_|2 ^DC^_t_|, in which DC_t_ was defined as the subtraction of the C_t_ value of the miR-630 from the C_t_ value of internal control U6 small nuclear RNA, respectively.

### Statistical analysis

SPSS 15.0 software (SPSS Inc., Chicago, IL, USA) was used for statistical analysis. All values are expressed as the Mean ± SEM of at least three separate experiments, using a two-tail Student’s *t* test to carry out comparisons of two independent groups. *P* value of < 0.05 was considered to be statistically significant. The AUC was used to compare the ability of miR-630 to discriminate patients with “HO” from those with other processes leading to bone formation. The optimal cut-points of miR-630 for indicating patients with HO were determined on the basis of the maximal of the Youden index (sensitivity plus specificity minus 1) with approximately equal weight being given to sensitivity and specificity.

### Study approval

This study was conducted according to the principles expressed in the Declaration of Helsinki. The clinical study and all mice experiments were reviewed and approved by the ethical committee of Shanghai Jiao Tong University Affiliated Sixth People’s Hospital. All participants in this study or their parents or legal guardians have signed written informed consent for the collection of samples and subsequent analyses.

## Additional Information

**How to cite this article**: Sun, Y. *et al.* MiR-630 Inhibits Endothelial-Mesenchymal Transition by Targeting Slug in Traumatic Heterotopic Ossification. *Sci. Rep.* **6**, 22729; doi: 10.1038/srep22729 (2016).

## Supplementary Material

Supplementary Information

## Figures and Tables

**Table 1:** Characteristics of subjects enrolled in.

	HO	Ctrl1	Ctrl2
N	146	292	292
Age*	34.6 ± 12.0	33.8 ± 12.1	34.1 ± 12.0
Sex(M/F)	93/53	186/106	186/106
% bone fraction on the right side	50.7	50.7	50.7
Serum miR-630 at baseline*	0.93 ± 0.03	2.58 ± 0.12	2.51 ± 0.09
Serum miR-630 at 3-month follow-up*	2.09 ± 0.09	2.50 ± 0.14	2.49 ± 0.11
Serum miR-630 at 1-year follow-up*	2.69 ± 0.12	2.66 ± 0.14	2.82 ± 0.16

^*^Data were presented as Mean ± SEM. *P* value at baseline < 0.01; paired *t* test.
