# Supplementary material for: MiR-630 Inhibits Endothelial-Mesenchymal Transition by Targeting Slug in Traumatic Heterotopic Ossification
Source: Sci Rep. 2016 Mar 4;6:22729. doi: 10.1038/srep22729 (PMC4778133; doi:10.1038/srep22729)
Supplement: Supplementary Information [file srep22729-s1.pdf]

MiR-630 Inhibits Endothelial-Mesenchymal Transition by Targeting Slug in Traumatic Heterotopic Ossification

Yangbai Sun[1,*], Jiangyu Cai[1,*], Shiyang Yu[1], Shuai Chen[1], Fengfeng Li[1#], Cunyi Fan[1#].

[1]. Department of Orthopaedics, Shanghai Jiao Tong University Affiliated Sixth People's Hospital, 600 Yishan Road, Shanghai, 200233, China

***** Yangbai Sun and Jiangyu Cai contributed equally to this article.

# For correspondence: fengmale@mail.sh.cn (Fengfeng Li);

fancunyi888@foxmail.com (Cunyi Fan)

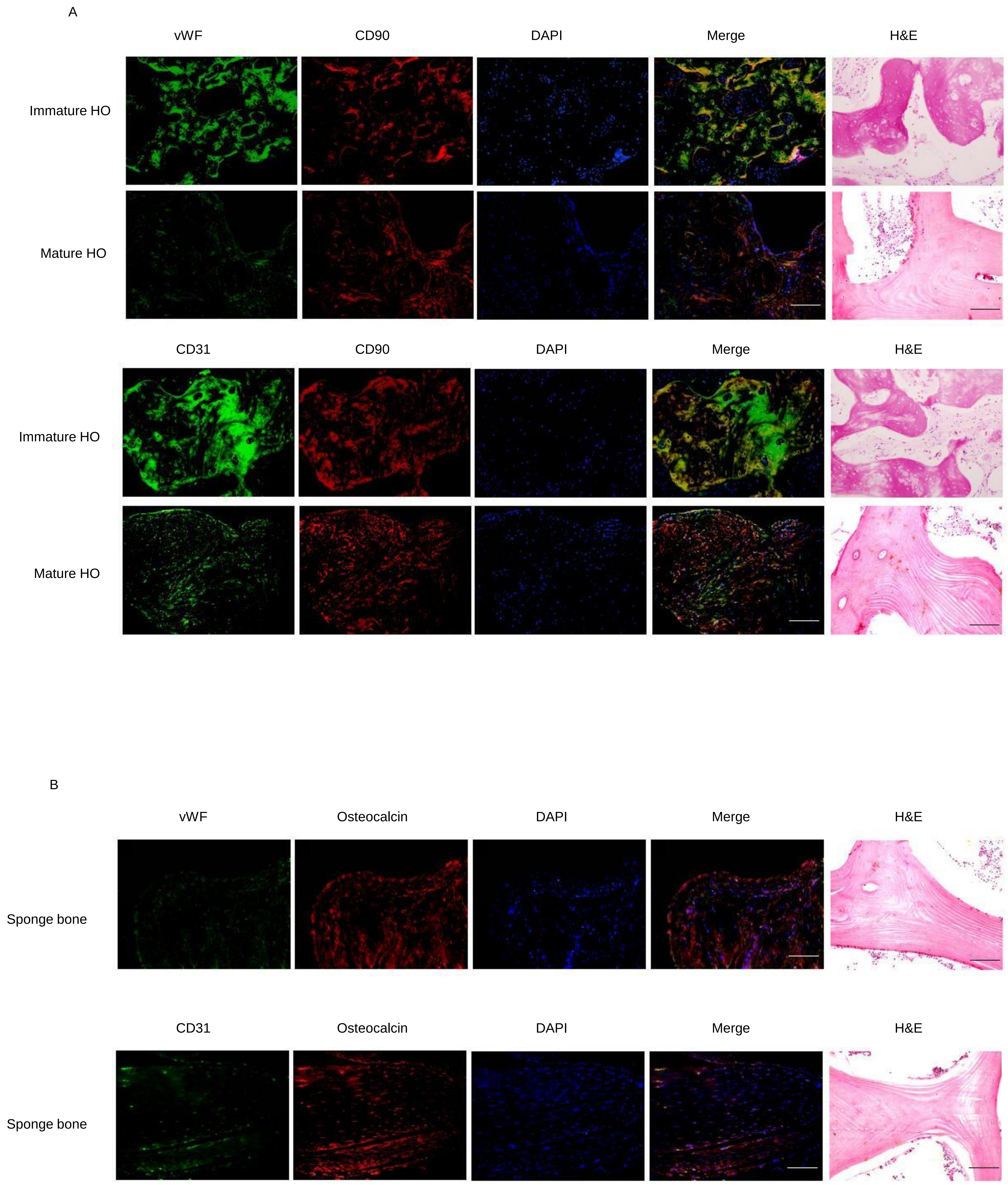

Supplementary Figure 1. (A) Immunofluorescence analysis of HO tissues from operation of trauma-induced HO patients was performed using the endothelial markers vWF, CD31 and the mesenchymal marker CD90. H&E staining was performed for the new bone of HO structure and cell composition. Scale bar, 50 μm. (B) Immunofluorescence analysis of endothelial markers vWF, CD31 and the osteoblast marker osteocalcin and H&E staining was performed for analyzing sponge bone as negative control. Scale bar, 50 μm.
